# Aquaporin 11, a regulator of water efflux at retinal Müller glial cell surface decreases concomitant with immune-mediated gliosis

**DOI:** 10.1186/s12974-016-0554-2

**Published:** 2016-04-23

**Authors:** Cornelia A. Deeg, Barbara Amann, Konstantin Lutz, Sieglinde Hirmer, Karina Lutterberg, Elisabeth Kremmer, Stefanie M. Hauck

**Affiliations:** Experimental Ophthalmology, Philipps University of Marburg, Baldingerstrasse, 35033 Marburg, Germany; Department of Veterinary Sciences, Institute of Animal Physiology, Ludwig-Maximilians University, Veterinärstr. 13, 80539 München, Germany; Helmholtz Zentrum München—German Research Center for Environmental Health (GmbH), Institute for Molecular Immunology, Marchioninistraße 25, 81377 München, Germany; Department of Protein Science, Helmholtz Zentrum München—German Research Center for Environmental Health (GmbH), Ingolstädter Landstr. 1, 85764 Neuherberg, Germany

**Keywords:** AQP11, AQP4, AQP5, Water efflux, Cell volume regulation, Water channel, Superaquaporin, Autoimmune uveitis, Retinal inflammation

## Abstract

**Background:**

Müller glial cells are important regulators of physiological function of retina. In a model disease of retinal inflammation and spontaneous recurrent uveitis in horses (ERU), we could show that retinal Müller glial cells significantly change potassium and water channel protein expression during autoimmune pathogenesis. The most significantly changed channel protein in neuroinflammatory ERU was aquaporin 11 (AQP11). Aquaporins (AQP, 13 members) are important regulators of water and small solute transport through membranes. AQP11 is an unorthodox member of this family and was assigned to a third group of AQPs because of its difference in amino acid sequence (conserved sequence is only 11 %) and especially its largely unknown function.

**Methods:**

In order to gain insight into the distribution, localization, and function of AQP11 in the retina, we first developed a novel monoclonal antibody for AQP11 enabling quantification, localization, and functional studies.

**Results:**

In the horse retina, AQP11 was exclusively expressed at Müller glial cell membranes. In uveitic condition, AQP11 disappeared from gliotic Müller cells concomitant with glutamine synthase. Since function of AQP11 is still under debate, we assessed the impact of AQP11 channel on cell volume regulation of primary Müller glial cells under different osmotic conditions. We conclude a concomitant role for AQP11 with AQP4 in water efflux from these glial cells, which is disturbed in ERU. This could probably contribute to swelling and subsequent severe complication of retinal edema through impaired intracellular fluid regulation.

**Conclusions:**

Therefore, AQP11 is important for physiological Müller glia function and the expression pattern and function of this water channel seems to have distinct functions in central nervous system. The significant reduction in neuroinflammation points to a crucial role in pathogenesis of autoimmune uveitis.

## Background

Autoimmune uveitis is a neuroinflammatory disease that is characterized by recurrent remitting episodes of intraocular inflammation [1]. Targets of the immune attacks, that are mainly carried out by autoaggressive T cells, are retinal proteins [2, 3]. Since the retina belongs to the central nervous system, every damage of its architecture means irreversible destruction and subsequently impairs visual function. Main actors from immune system are T helper cells (CD4+) [4]. Additionally, B cells participate through autoantibodies [2] and cells of the innate immune system also contribute to pathophysiology [5]. To current knowledge, there are T helper cell subsets from Th1 and Th17 pathways involved in this progressive, organ-specific autoimmune disease pathology [4, 6–8].

The role of retinal cells itself in sight-threatening complications during inflammation has lately gained more interest, since it was shown that intraocular inflammation is the result of interplay between cells of the retina and cells of the immune system. Invasion of retina by immune cells and subsequent reversible destruction of this central nervous system-associated tissue is also critically dependent on the response of cells from the retina itself [9]. Without correspondent reaction of retinal cells, autoaggressive immune cells alone will not lead to invasion and intraocular inflammation [9]. We identified retinal Müller cells, the dominant macroglial cells of the retina as decisive cells in uveitis immune pathology [10, 11].

Through proteome analyses of changed membrane proteins in ERU retina, we identified numerous differentially expressed proteins in disease [12]. A cluster of changed channel membrane proteins was identified, and we subsequently verified and further characterized respective differential expression [10]. Interestingly, mostly retinal Müller glial cells were related to changed abundance and distribution of potassium channels (Kir 2.1 and 4.1), as well as two aquaporins (4 and 5) [10].

Aquaporins are integral membrane proteins with 13 members to date [13]. Aquaporins generally control water transport and transcellular water flow and are important regulators of cellular volume [14]. A rapid change of cellular volume always provokes a reaction by aquaporins [14]. Aquaporins can be assigned to three different functional groups, the first comprises orthodox aquaporins, that primarily transport water (AQP0, 1, 2, 4, 5, 6, 8) [13]. The second group contains aquaglyceroporins (AQP3, 7, 9, 10) that passage water and neutral solutes [13]. The third group encloses only two members so far, AQP11 and 12; both are only distantly related to the other aquaporins and have low amino acid sequence similarities to the proteins of both other groups [13].

Interestingly, we found AQP11 as the most significantly regulated channel protein in enriched membrane fraction of ERU retina. AQP11 is the latest member of the aquaporin family, was shown to be localized to the plasma membrane [15], and is presumed to have an important role in endoplasmatic reticulum (ER) function [16]. AQP11 knockout mice die due to a malfunction of their kidneys with degeneration of proximal tubule cells [17]. Further, AQP11 was shown to prevent glucose-induced oxidative stress in proximal tubules [18]. Besides other organs, AQP11 was also shown to be expressed in the eye [19], but in general, the function of AQP11 is still largely unknown [13]. Since biological functions of AQP11 remain to be elucidated and our finding was the first evidence pointing to an important role of AQP11 in a spontaneously occurring neuroinflammatory disease involving the retina, we were interested to gain further insight into the role of AQP11. Retinal edema is a severe complication of recurrent uveitis [20]. Since Müller glial cells are important regulators of intraretinal water flow [21] and aquaporins provide one important mechanism for water movement, the goal of this study was to test the hypothesis that AQP11 function plays a critical role in the context of inflammation and osmotic stress in the eye.

## Methods

### Specimen

For this study, a total of 78 healthy controls and 33 ERU eyes and were used (Western blots, 11 controls, 12 ERU; immunohistology, 11 controls, 21 ERU; immune cytology, 46 controls). ERU specimens were derived from horses that were treated in the equine clinic and diagnosed with ERU according to clinical criteria as described [22]. All eyes used for this study were from animals that were euthanized due to causes unrelated to this study (control and ERU eyes). These causes were evenly distributed in both groups (controls and ERU cases) and did not involve systemic inflammatory or neurological conditions (e.g., chronic degenerative lameness). Three ERU eyes were immediately enucleated after a therapeutical procedure (vitrectomy) because of death in general anesthesia. Removal of vitreous or the respective chronic diseases did not influence the retinal structure for this study. Collection and use of equine eyes from animals that were killed due to a research-unrelated cause was approved for purposes of scientific research by the appropriate board of the veterinary inspection office Munich, Germany (permit number: 8.175.10024.1319.3). All animals were treated according to the ethical principles and guidelines for scientific experiments on animals according to the ARVO statement for the use of animals in Ophthalmic and Vision Research. No experimental animals were involved in this study.

### Sample preparation

Immediately after enucleation, retinas were isolated from the eyeballs as described [10]. For the preparation of lysates for western blots, retinas were immediately stabilized with protease inhibitors (Roche, Mannheim, Germany), homogenized, lyophilized ,and subsequently dissolved in lysis buffer (9 M urea, 2 M thiourea, 65 mM DTT, 4 % CHAPS). Protein content was determined using the Bradford assay (Sigma-Aldrich, Taufkirchen, Germany).

For the preparation of primary retinal Müller glial cells for cell culture experiments, these cells were separated from the retinal tissue as previously described [23]. In brief, retinal tissue fragments were incubated at 37 °C with papain for 30 min (Roth, Karlsruhe, Germany). Papain activity was induced with 1.1 μmol EDTA, 0.067 μmol mercaptoethanol, and 5.5 μmol cysteine-HCL for 30 min at 37 °C prior to use. The reaction was stopped by adding DMEM with 10 % fetal calf serum (PanBiotech, Aidenbach, Germany). After addition of desoxyribonuclease I (Sigma-Aldrich, Taufkirchen, Germany) and trituration, the cells were collected by centrifugation (800 *g*, 10 min, RT), resuspended in DMEM with 10 % FCS and 1 % penicillin/streptomycin (all PanBiotech, Aidenbach, Germany), and seeded into 25-cm^2^ flasks (Sarstedt, Nümbrecht, Germany). After attachment for 24 h, cells were purified through repeated vigorous washing and removal of supernatant containing non-attached neuronal cells.

For immunohistochemical stainings, posterior eyecups were sliced into pre-assigned pieces as described [24], fixed in Bouin’s solution (Applichem, Darmstadt, Germany), and dehydrated in a series of alcohols. Resulting tissue blocks were embedded in paraffin, sectioned (8 μm), and mounted on coated slides (Superfrost Plus, Medite, Burgdorf, Germany). For validation of AQP11 antibody, we further used sections from negative control mouse and rat eyes and mouse kidney from earlier experiments. These specimens were also fixed in Bouin’s solution.

### Antibodies

A novel mouse monoclonal antibody clone 8H9 to AQP11 was generated towards a linear 12mer epitope of horse AQP11 in a widely conserved sequence. The antibody is cross-reactive to human, mouse, and rat AQP11; its isotype is mouse IgG1. For detection of Müller glial cells, we additionally used mouse antiglutamine synthase (GS) antibody (BD Biosciences, Heidelberg, Germany) and for detection of reactive gliosis, we used rabbit antiglial fibrillary acidic protein (GFAP; Sigma-Aldrich, Taufkirchen, Germany) antibody. As loading control and for normalization, we used mouse antibeta-actin antibody (Sigma-Aldrich, Taufkirchen, Germany) or mouse anti-GAPDH antibody (Merck-Millipore, Darmstadt, Germany). For inhibition of AQP4, we used mouse anti-AQP4 (Santa Cruz, Heidelberg, Germany); for blockage of AQP5, we used rabbit anti-AQP5 (Chemicon, Darmstadt, Germany) antibodies. AQP4 and 11 inhibitions through anti-AQP4 and AQP11 antibodies in cell culture experiments were controlled with mouse IgG isotype control antibodies; AQP5 inhibition was controlled with rabbit IgG. As secondary antibodies, anti mouse IgG HRP antibody was used for western blots and conventional immunohistochemical staining, and goat anti rabbit IgG Alexa 647 1:500, goat anti mouse IgG1-FITC 1:200, or goat anti mouse IgG coupled to Alexa 546 1:500 (all from Invitrogen, Karlsruhe, Germany) were taken for immunohistochemistry and cytology.

### Quantification of AQP11 expression with western blots

PVDF membranes (GE Healthcare, Freiburg, Germany) were loaded with equal total protein amounts from retinal lysates after separation by SDS-PAGE (12 % gels) and semidry blotting. To prevent unspecific binding, membranes were blocked with 4 % bovine serum albumin for 1 h at room temperature. Blots were then incubated with primary antibody (undiluted supernatant of novel anti-AQP11 clone 8H9) or with anti-beta actin antibody (dilution 1:50,000) or with anti-GAPDH antibody (dilution 1:500). After incubation with secondary HRP-coupled antibody for 1 h at room temperature, signals were detected by enhanced chemiluminescence on X-ray films (Fuji; Christiansen, Planegg, Germany).

Films were scanned on a transmission scanner, and quantification of western blot signals by densitometry was performed using ImageQuantTL software (GE Healthcare, Freiburg, Germany). AQP11 abundances were normalized to beta actin to ensure comparison of equally loaded cells, and subsequently, expression differences between ERU cases and controls were statistically analyzed using Student’s *t* test. Differences in protein expression were considered significant, if *p* value was ≤0.05.

### Analyses of AQP11 expression in healthy and diseased eyes

For detection of AQP11 in eyes from our paraffin-embedded tissue bank of physiological control eyes and ERU cases from various stages of disease, heat antigen retrieval was performed at 99 °C for 15 min in 0.1 M EDTA-NaOH buffer (pH 8.0). For prevention of unspecific antibody binding, sections were initially blocked with 1 % BSA in TBS-T and 5 % normal goat serum. Blocking serum was chosen according to the species the secondary antibody was produced in. Cell nuclei were counter-stained with DAPI (Invitrogen, Karlsruhe, Germany) or hematoxylin. For multiple labeling, blocking steps (ProteinBlock; DakoCytomation, Hamburg, Germany) were applied before every antibody incubation. For fluorescence triple labeling, sections were sequentially incubated with primary antibodies (AQP11 4 °C overnight; glutamine synthase 1:1500 and GFAP 1:1000 for 3 h at RT), always followed by respective secondary antibodies (30 min at RT). Finally, the sections were mounted with glass coverslips using fluorescent mounting medium (Carl Roth, Karlsruhe, Germany). Fluorescent images were recorded with Axio Imager M1 or Z1 and software Axio Vision 4.6 (Zeiss, Göttingen, Germany). Sections for the conventional immunohistology were stained with Vector VIP staining kit (Biozol, Eching, Germany) and recorded with Leica DMR microscope (Leica, Wetzlar, Germany). For all stainings, negative controls were performed with isotype controls of irrelevant specificity. To assess epitope specificity of our novel AQP11 antibody, we performed preincubation experiments with rising concentrations (1, 10, 100 μg/ml antibody supernatant) of the AQP11 immunization peptide with the AQP11 antibody (for 30 min at 37 °C). As a negative control, we used even concentrations of irrelevant CD3 peptide for preincubation. Binding capacity of preincubated antibodies was then analyzed with fluorescence immunohistochemistry, and intensity was compared to straight AQP11 antibody staining.

### Functional analyses of AQP11 in primary retinal Müller glial cells

To investigate AQP11 function in primary retinal Müller glial cells, we seeded 1 × 10^4^ cells per well in sterile multichamber slides (Millicell EZ 8-well glass slides, Merck Millipore, Darmstadt, Germany). Cells were then challenged with hyperosmolar (DMEM with 30.8 mmol NaCl), hypoosmolar (DMEM diluted with aqua dest. 1:5), or hyperglycemic (DMEM with 25 mmol glucose) conditions for 30 min. After thorough washing, cells were fixed with 2 % PFA for 30 min on ice. Then, cells were stained with both hematoxylin and eosin (Roth, Karlsruhe, Germany). Images were recorded with either Leica DMR (40× objective magnification) or Axio Vision Imager M1 (40×), and resulting images were imported into Adobe Photoshop software for further analyses. Respective measurements were used to calculate and compare cell and organelle sizes between the different conditions. To identify the role of AQP11 in regulation of cell size in these states, we blocked AQP11 through preincubation of cells with our monoclonal antibody and compared the response to inhibition with respective antibodies to AQP4 (Santa-Cruz, Heidelberg, Germany, 1:50) or AQP5 (Merck-Millipore, Darmstadt, Germany, 1:200). As negative control, we used preincubation with isotype control antibodies. Cell size areas and that of nuclei of each condition were measured. A total of 46 biological replicates with 10 cells each were comprised in the statistical analysis per approach. Mann-Whitney test was performed for comparison of cell size in physiological condition and after osmotic challenge. The results were regarded as significant, if the *p* value was ≤0.05.

## Results

### Aquaporin 11 is the most significantly regulated channel protein in uveitic retina

In an earlier study, we detected differentially regulated candidates in membrane protein-enriched fractions of healthy retina compared to ERU [12]. Among channel proteins, aquaporin 11 (AQP11) was the protein with most changed abundance in disease (Table 1). Since AQP11 is a protein which function is still discussed and this was the first association of this protein with an autoimmune disease, we were interested to further characterize the meaning of AQP11 downregulation for ERU pathogenesis.

**Table 1:** Identification and regulation of AQP11 protein in healthy compared to ERU retina as assessed by mass spectrometry

Protein name^a^	Accession no.^b^	Confidence score^c^	Peptide count^d^	Anova^e^	Ratio control/ERU^f^
Aquaporin-11	ENSECAP00000015158	241	3	*p* ≤ 0.001	4.56

^a^Protein name

^b^Accession number as listed on Ensembl database (http://www.ensembl.org/index.html)

^c^Confidence score as given in Mascot

^d^Number of peptides used for identification

^e^Significance of differential expression of proteins in healthy cases compared to control by Anova

^f^Regulation factor

### Establishment of novel antiaquaporin 11 antibody

For characterization of AQP11 function in equine retina, we first generated an antibody suited for a respective AQP11 detection. Clone 8H9 proved to identify equine AQP11 (controlled by immune precipitation with equine retinal lysate and subsequent detection of AQP11 by mass spectrometry, data not shown) with all techniques applied for this study (western blots, immunohistochemistry/cytology and blocking of AQP11).

### Aquaporin 11 is exclusively expressed at retinal Müller glial cells in the retina

**Fig. 1:**
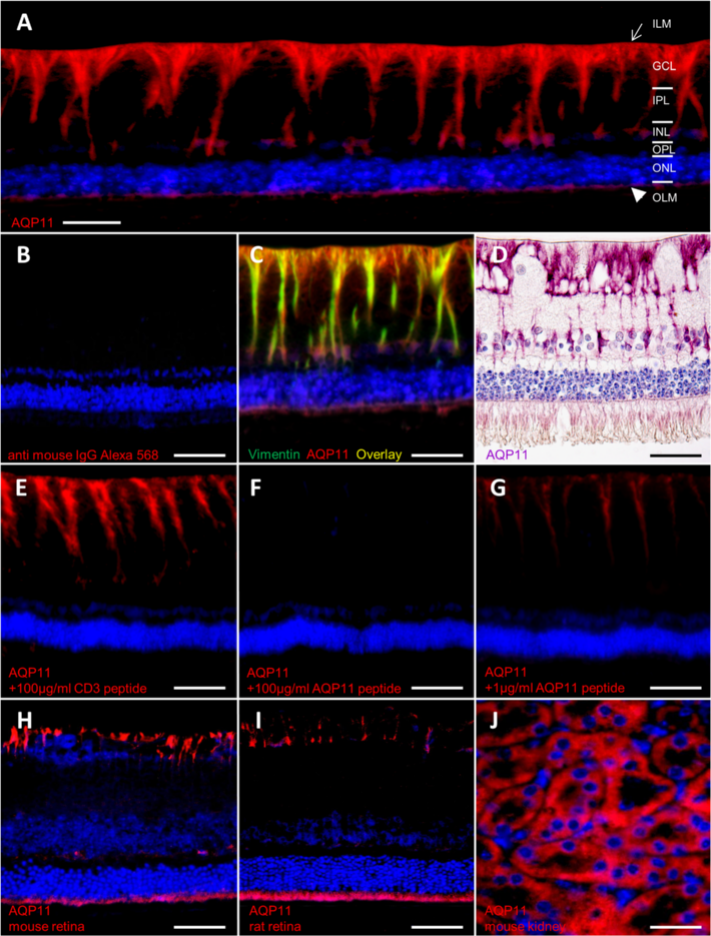
Assessment of AQP11 expression with immunohistochemistry, scale bars = 20 μm. **a** Expression in equine retina. AQP11 (*red*) was specifically expressed at retinal Müller glial cells, reaching from endfeet in ganglion cell layer to outer limiting membrane; *ILM* inner limiting membrane (*arrow*), *GCL* ganglion cell layer, *IPL* inner plexiform layer, *INL* inner nuclear layer, *OPL* outer plexiform layer, *ONL* outer nuclear layer, *OLM* outer limiting membrane (*arrowhead*). **b** Negative control, incubated only with secondary antibody anti mouse IgG Alexa 568. **c** Overlay of Müller glia marker vimentin (*green*) and AQP11 expression (*red*) in equine retina results in yellow color. **d** Müller glia cell membrane localization of AQP11 (*violet*) stained with fine-grained resolution. **e** Preincubation of AQP11 antibody with 100 μg/ml irrelevant peptide did not interfere with antibody binding, AQP11: *red*. **f** Preincubation with 100 μg/ml AQP11 immunization peptide completely blocked antibody binding. **g** Preincubation with 1 μg/ml AQP11 immunization peptide partly blocked antibody binding. **h** Müller glia endfeet of mouse and **i** rat retina express AQP11 (*red*). **j** AQP11 (*red*) expression in positive control tissue mouse kidney

In retinal sections, we subsequently detected AQP11 expression specifically at retinal Müller glial cells (Fig. 1a, AQP11 red, endfeet located in ganglion cell layer = GCL, arrow). AQP11 was distributed all over the cell, with strong signals at endfeet, ending with a fainter expression at outer limiting membrane (=OLM, Fig. 1a, arrowhead). Apart from RMG cells, no other structures of the retina were positive for AQP11, as tested for example for endothelial cells by costaining with Lycopersicon esculentum lectin, a vascular endothelial marker in horses [25], which did not result in an overlap. In contrast, double staining with vimentin, a Müller glia cell marker (Fig. 1c, green), resulted in clear overlap with AQP11 (Fig. 1c, red) expression (Fig. 1c, yellow color). A high magnification image with peroxide color staining enabled the exact localization of AQP11 along Müller glial cell membrane (Fig. 1d, AQP11: violet). Preincubation of AQP11 antibody with immunization peptide, but not with irrelevant peptide dose dependently omitted binding of antibody to AQP11 (Fig. 1e, preincubation with 100 μg/ml irrelevant peptide, 1F: preincubation with 100 μg/ml AQP11 peptide, 1G: 1 μg/ml AQP11 peptide). AQP11 prominently labeled the entire Müller glia cells in horse retina, expanding along Müller cell bodies until the outer limiting membrane. This is different to the description in human retina, where AQP11 was primarily detected at the inner limiting membrane formed by Müller cell endfeet [26]. However, both species present with Müller cell-specific AQP11 expression as observed by immunostaining. We have observed before that other marker proteins (GFAP, Glutamine synthase) label the entire structure of Müller cells in normal horse retina [2, 11], while in other species as mouse [27], rat [2], and human [28], only marginal staining close to the endfeet is visible. Therefore, AQP11 also labels Müller glia cells in mouse (Fig. 1h, AQP11: red) and rat retina (Fig. 1i, AQP11: red). AQP11 was also detectable in mouse kidney, a positive control tissue for AQP11 expression (Fig. 1j, AQP11: red).

### Expression of AQP11 profoundly decreases in recurrent uveitis

**Fig. 2:**
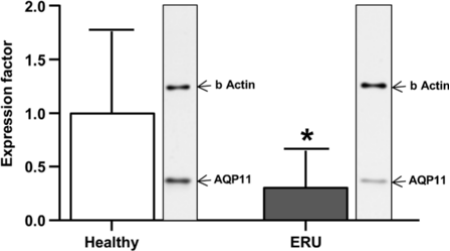
Marked decline of AQP11 protein expression in ERU retinas, scale bars = 20 μm. Western blot analyses demonstrated that in inflamed retinas; AQP11 abundance was reduced to one third (31 ± 33 %) of physiological quantity (*p ≤ 0.05). *White bars* represent quantified expression of AQP11 in control retinas (*n*=), g*rey bars* of ERU retinas (*n*=). Entire blot strips of retinal proteins of representative control horse and ERU case beside respective bars. The blots were first incubated with anti-AQP11 antibody (lower band) and then with beta actin antibody (upper signal) for normalization

**Fig. 3:**
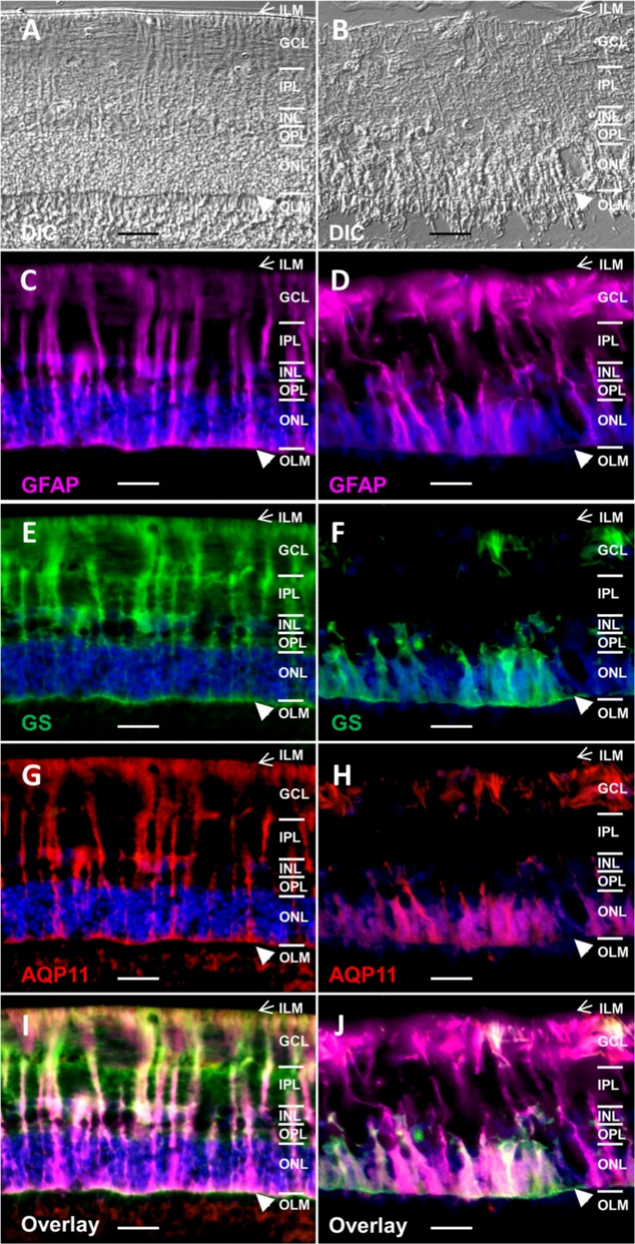
AQP11 expression reduction is associated with gliotic changes in ERU retinas, scale bars = 20 μm. *ILM* inner limiting membrane (*arrow*), *GCL* ganglion cell layer, *IPL* inner plexiform layer, *INL* inner nuclear layer, *OPL* outer plexiform layer, *ONL* outer nuclear layer, *OLM,*outer limiting membrane (*arrowhead*). In uveitis, AQP11 expression decreases secondary to gliosis. **a** Physiological retina. **b** ERU. **c** GFAP (*violet*) expression in healthy Müller glial cells. **d** Upregulation of GFAP (*violet*) in gliotic Müller cells in ERU. **e** GS (*green*) expression in control Müller cells. **f** Reduction of GS (*green*) in gliotic Müller cells in ERU. **g** Physiological AQP11 (*red*) expression in control retina. **h** Decline of AQP11 contrary to GFAP upregulation in ERU indicates diminished AQP11 in gliotic Müller cells; overlay image of GFAP (*violet*), GS (*green*), and AQP11 (*red*) in **i** physiological control retina and **j** ERU retina. **i** In healthy retina, Müller cell markers and AQP11 widely colocalize (triple overlay = *white color*). **j** In contrast, in ERU, gliosis dominates with upregulation of GFAP (*violet*) and concomitant loss of GS (*green*) and AQP11 (*red*), (triple overlay = *white color*)

We next verified significant reduction of AQP11 expression in ERU cases, since our initial finding of AQP11 decline as most significantly reduced channel protein in ERU prompted us to analyze AQP11 function in retina. Quantification of AQP11 in healthy retinas versus ERU cases confirmed profound reduction of AQP11 expression in diseased retinas to 32 % of physiological expression (Fig. 2, white bar: controls, grey bar: ERU cases). Triple staining of healthy (Fig. 3a) and uveitic retinas (b, differential interference contrast images) with gliosis markers GFAP (Fig. 3c, d, violet) and GS (Fig. 3e and f, green) revealed typical gliosis reaction of retinal Müller glial cells in inflammatory condition. GFAP was clearly upregulated in ERU (Fig. 3d) concomitant with lower abundance of GS in these cells (Fig. 3f). Interestingly, the decrease of AQP11 followed GS expression pattern in all tissues analyzed (Fig. 3h, representative image of 21 ERU eyes, AQP11: red). Therefore, we conclude that reduction of AQP11 is indicative of gliotic Müller cells from our analyses.

### AQP 11 regulates cell volume through water efflux in Müller glial cells

**Fig. 4:**
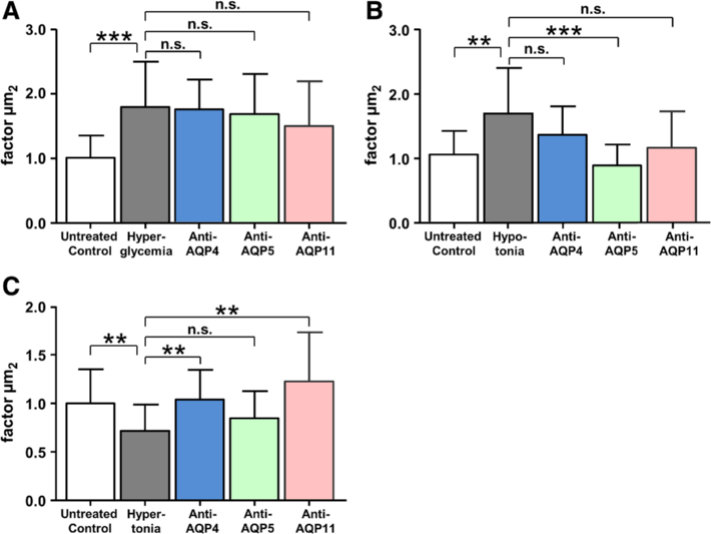
AQP11 plays a role in cell volume regulation of Müller glial cells. Hyperglycemic (**a**), hypotonic (**b**), and hypertonic (**c**) extracellular environments led to significant respective cell volume regulations. Inhibition of AQP4 (*blue bar*), AQP5 (*green bar*), or AQP11 (*red bar*) did not change cellular response under hyperglycemic conditions (Fig. 4a). Under hypotonia, only blockage of AQP5 rescued the cells from swelling (Fig. 4b, *green bar*). In contrast, inhibition of AQP4 and AQP11 channels did not change reaction in hyperglycemia and hypotonia (Fig. 4a, b) but significantly prevented reduction of cell size in hypertonic condition (Fig. 4c, *blue bar* AQP4, *red bar* AQP11). **p ≤ 0.01; ***p ≤ 0.001

**Fig. 5:**
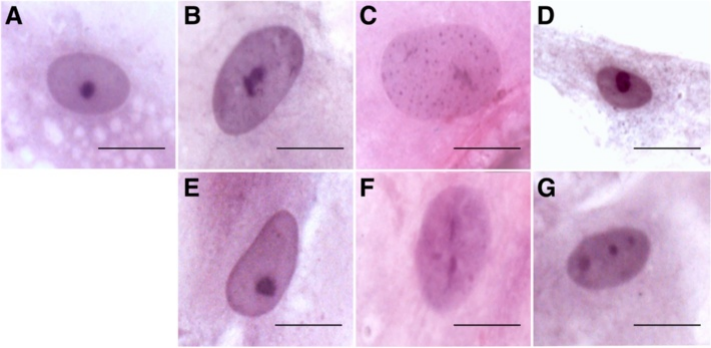
Blockage of AQP11 prevents shrinking of cells in hypertonic environment, scale bars = 20 μm. Primary native retinal Müller glial cells (**a**) were incubated in different osmotic stress conditions (**b** hyperglycemia, **c** hypotonia, **d** hypertonia) and initiated cell volume regulation. **e**–**g** Respective osmotic stress condition of cell above, but with inhibition of AQP11 channel through preincubation with AQP11 antibody. Only shrinkage under extracellular hypertonia (**d**, **g**) was inhibited with anti-AQP11 antibody preincubation (**e**–**g**). Staining of cells: H&E

Since the function of AQP11 is still under debate in many animals and different cell types, we were interested to analyze AQP11 function in native equine Müller glial cells. Therefore, we analyzed cell volume regulation of these cells under different osmotic challenges and created hyperglycemic, hypotonic, and hypertonic environments in cell culture. To assess AQP11 function in these conditions, we blocked AQP11 function with our novel monoclonal antibody and compared the effects to inhibition of AQP4 and 5, respectively. All applied settings led to a significant change in cell areas (Fig. 4). Hyperglycemic (Fig. 4a) and hypotonic environments (Fig. 4b) led to significant regulatory volume increase of Müller glial cells (Fig. 4a: factor 1.8, ***p ≤ 0.001, 4b: factor 1.7, **p ≤ 0.01) and hypertonic environment to a regulatory volume decrease (Fig. 4c: factor 0.7, ** = *p* ≤ 0.01). Blockage of AQP11 water channel did not prevent the swelling of cells under hyperglycemic and hypotonic conditions (Fig. 4a, b, red bars). In contrast, in hypotonic environment, only inhibition of AQP5 averted the increase of cell nuclei (Fig. 4b, green bar). But in hypertonic condition, shrinking of cells was totally abolished through inhibition of AQP11 channels (Fig. 4c, red bar, **p ≤ 0.01). Inhibition of AQP4 was also selectively efficient to prevent cell shrinking in hypertonia (Fig. 4c, blue bar, **p ≤ 0.01), whereas blockage of AQP5 did significantly change cellular reaction to hypotonic environment (Fig. 4b, green bar, ***p ≤ 0.001). Therefore, AQP5 seems to have another direction in cell volume regulation of Müller glia cells, whereas AQP4 and 11 seem to have similar functions in cell volume control. Morphology of Müller glial cells changed accordingly (Fig. 5). Hyperglycemic and hypotonic media led to swelling of cell bodies (Fig. 5b, c) and hypertonia to cell volume decrease (Fig. 5d). Only the latter phenotype was rescued by preincubation of cells with anti-AQP11 antibody, which blocked the respective water channel (Fig. 5e–g). Control incubation with isotype antibody did not result in cell volume change (Fig. 5b–d).

## Discussion

In addition to potassium (Kir 2.1 and 4.1) and water (AQP4 and 5) channel proteins found differentially expressed in retinal membrane proteins of horses with ERU by us [12], we detected AQP11 as the most significantly downregulated candidate in pathological condition of ERU. AQP11 is a very interesting water channel protein because it is quite different to most of the other members of aquaporin family proteins [13]. An exception is AQP12 that shares similarities with AQP11, at least regarding the unusual N-terminal sequence with an NPA (asparagine-proline-alanine) motif and low homology to the other aquaporins [13, 16]. AQP11 was primarily described as not only most abundantly expressed in testis and to a lower extent, but also prominent in the kidney, liver, and brain [29]. AQP11 knockout mice die early because of renal failure and retarded growth [30]. An ocular phenotype was not described in these mice so far [30]. Nevertheless, all aquaporins were reported to be expressed in the eyes of several mammals analyzed [31]. AQP11 is expressed at Müller glia endfeet in the eyes of humans [26]. In this study, we could clearly confirm AQP11 expression for Müller glia endfeet, but additionally, AQP11 is expressed throughout entire Müller glia plasma cell membrane in horse retina (Fig. 1a, c, d). After we identified AQP11 as the most pronounced downregulated channel protein in retinal membrane protein fraction (Table 1) in a differential proteomics experiment analyzing physiological retina versus autoimmune diseased retina in a spontaneous horse model [12], we were quite interested in the exact cellular localization and function of this less good characterized member of the aquaporin family [32]. Therefore, we developed respective antibody to horse AQP11 that allows detection of AQP11 in paraffin-embedded sections (Figs. 1 and 3) and western blots (Fig. 2) and to block AQP11 function in native Müller glial cells (Figs. 4 and 5). This was important because tissue localization and functional studies about unorthodox aquaporins in general are currently hampered because of poor-quality antibodies available [17, 33–35]. Our novel monoclonal antibody is well suited to analyze AQP11 expression in immunohistochemistry and will probably aid other researchers interested in characterization of AQP11 in other animals or organs, since there is a reported lack of a good antibody suited for immunohistochemistry so far (positive stainings with antibody 8H9 in other species/tissues: Fig. 1h: mouse retina, i: rat retina, j: mouse kidney). With our novel tool, we could confirm marked reduction of AQP11 expression in a spontaneous model of retinal inflammation (ERU) to one third of physiological amount in retinas (Fig. 2). AQP11 was selectively expressed in retinal Müller glial cells in the retina of horses (Figs. 1a, c and 3g) and especially declined (Fig. 3h) in the areas of Müller cell gliosis (Fig. 3d, f) characterized in situ through upregulation of GFAP and concomitant lower expression of glutamine synthase in ERU (Fig. 3d, f). Reduction of AQP11 expression in ERU (Fig. 3h) closely followed glutamine synthase expression in respective areas (Fig. 3f, overlay j). Since Müller glial cells are still present in these regions, AQP11 reduction is not secondary to loss of expression sites, but seems to be related to inflammation-associated gliosis (Fig. 3). To our knowledge, this is the first link of AQP11 to a spontaneous autoimmune disease and in neuroinflammation. Because AQP11 function is still debated and ranges from it have no water permeability ability at all, as shown with Xenopus oocytes expressing AQP11 [15] or has low but normal water channel activity with mercurial sensitivity, we were interested to test the influence of AQP11 to water permeability in Müller cells. Our results clearly show that AQP11 channel is especially important during osmotic stress conditions and blockage of respective channel protein results in the rescue from cell shrinking in extracellular hypertonic conditions (Figs. 4 and 5). Inhibition of AQP4 showed the same results as application of anti-AQP11 antibody, a selective saving from shrinking in hypertonic milieu, whereas blockage of AQP5 positively influenced cellular behavior in hypotonic environment (Fig. 4). Further, we could clearly show that AQP11 is expressed at Müller glial plasma cell membrane (we additionally confirmed the expression of AQP11 at Müller glia endfeet in mouse and rat retina). AQP11 expression was detected intracellularly [31, 32] in many organs of other species, especially at rough ER [36, 37]. Interestingly, we could not discover AQP11 at Müller glial cell ERs, as tested with ER markers calreticulin and calnexin in double stainings with AQP11, nor with various other cell organelle markers tested (e.g., for mitochondria, ribosomes, Golgi a. o., analyzed with apotome) or other cell types like endothelial cells (costaining with specific plant lectins). Therefore, we conclude that AQP11 is preliminary expressed at the cell surface of Müller glial cells. This would indicate an interesting difference of AQP11 in various cell types. But so far, several discrepancies in AQP distribution have been reported for different cells and tissues, including its location within lung and airways, gut, and exocrine glands [38], underscoring the need for further analyses regarding cellular and subcellular distribution of AQP11. For example, localization of AQP11 at the plasma cell membrane remains controversial for other cells. Immunocytochemistry was used to show the expression of AQP11 on the cell surface of transfected Chinese hamster ovary cells [15], whereas other data indicated that AQP11 is not targeted to the plasma membrane and stays intracellularly [36]. In liver-specific AQP11 knockout mice, immunoblotting of membrane proteins from control mouse liver indicated AQP11 in this fraction, but exact cellular localization could not be investigated in this study because their antibody was not suited for immunohistochemistry [17]. Probably, this information can now be created with our novel antibody and therefore shed more light on the unclear biological function of this interesting membrane channel protein.

Most studies focused on the role of AQP11 in ER function so far. It was shown that disruption of AQP11 in knockout mice leads to apoptosis in kidney cells associated with upregulation of ER stress genes [39]. In Müller glial cells, AQP11 is expressed at cell surface and its significant reduction in ERU will therefore result in a change in transport function through plasma cell membrane. In our study with primary Müller glial cells, we could clearly show a function in cell volume regulation after osmotic stress to the cells. Blockage of AQP11 totally abolished the shrinking of treated cells (Figs. 4 and 5) like AQP4 did, indicating a similar function for both AQPs in outward water transport of retina. Redundant expression of different aquaporins was also shown for other tissues, e.g., of AQP3 and AQP4 in the basolateral plasma membrane of collecting duct principal cells, where they colocalize [38]. The functional meaning of such coexpression is not fully understood to date. Since these channels also transport some other compounds besides water, there could be some other functional differences. The data concerning the ability of AQP11 to transport water are conflicting, and there is a lack of knowledge about the permeability of AQP11 to other solutes [15, 40]. Therefore, the meaning of AQP4 and AQP11 coexpression und probable cofunction in the retina needs further investigation in our opinion.

We earlier observed in ERU retinas that the total amount of AQP4 increased, whereas AQP5 decreased [10]. But interestingly, AQP4 expression changed its localization in ERU from Müller cells to a strong circular expression in the outer nuclear layer and rarely in the inner nuclear layer, whereas its expression in Müller cell trunks almost disappeared [10]. Since our proteome analyses of healthy and ERU retinas did not indicate further AQPs besides 4, 5, and 11 in physiological or ERU condition [12], the question arises, how ERU retinas handle water control without these aquaporins. Probably, water flow is still controlled by AQP4 despite re-localization, although we cannot entirely rule out other pathways/channels [38] capable of water transport in retinal pathophysiology.

Previously, we already identified a loss of potassium (Kir 2.1 and 4.1) and water channels (AQP4 and 5) in gliotic Müller glial cells in ERU [10]. Retinal edema is a severe complication of late-stage ERU [20] and can be caused by swollen Müller glial cells [21]. Severe cases of gliosis induce the de-differentiation of Müller glial cells, followed by impairment of the ion and water homeostasis after downregulation of K+ channels [21]. Downregulation and changed expression of Kir channels already takes place in early stages of ERU [10]. Downregulation of these K+ channels can subsequently inhibit K+ release into the blood and interrupt the dehydrating K+ currents through Müller cells, which can increase the osmotical pressure within the cells. This results in an osmotic difference at the gliovascular interface which favors water inflow into the cells via still expressed aquaporin (especially AQP4) water channels [21]. Osmotic stress can then activate phospholipase A2 and arachidonic acid (or inflammation during ERU) and can further increase theinflux of Na+ ions and therefore create an osmotic driving force for water inflow into the cells, resulting in Müller cell swelling [21]. These mechanisms lead to significant dysregulation of retinal dehydration by Müller cells and to exacerbation of intra- and extracellular fluid accumulation [21]. The loss of AQP11 channels at Müller glia plasma cell membranes (Fig. 3) probably reduces the ability of the cells to reduce cell volume through outflow of water and therefore leads to cell swelling and subsequent fatal retinal edema not only in ERU but also other retinal diseases, e.g., diabetic macular edema.

The role of AQP11 could additionally be tested by administering recombinant AQP11 to the cells in the in vitro studies. Although we have recently established virus-based transduction protocols with the hope to overcome the minimal transfection efficiencies observed for primary Müller cells (from horse and pig), we so far only achieved low transduction rates with lentivirus (maximal 5 %) and consequently at this time, those experiments are not feasible. But in future studies, we plan to study the effect of adding AQP11 to the cells under different environmental conditions in vitro.

## Conclusions

Our finding of most downregulated water channel AQP11 in a retinal autoimmune disease is novel and very interesting in our point of view because it links this poorly characterized protein to a role in retinal Müller glial cell function in a spontaneous inflammatory eye disease. Further, we could assign AQP11 expression specifically to retinal Müller glial cell plasma cell membranes and demonstrate a function for cell volume regulation through outward water transport. Since AQP11 is also selectively expressed in brain endothelium that forms blood-brain barrier and is important for the prevention of brain edema [34], the expression pattern and function of this water channel seems to have distinct and important functions in the central nervous system. The finding of decreased abundance in the context with neuroinflammation in a spontaneous autoimmune disease indicates its relevance in immune pathogenesis.

### Main findings

- AQP11, an unorthodox water channel protein, is expressed at Müller glia plasma cell membrane
- AQP11 decreases in a spontaneous inflammatory retinal disease
- AQP11 probably is a channel for outward water transport from Müller glia

## References

[1] Degroote RL, Hauck SM, Amann B, Hirmer S, Ueffing M, Deeg CA (2014). Unraveling the equine lymphocyte proteome: differential septin 7 expression associates with immune cells in equine recurrent uveitis. PLoS One.

[2] Deeg CA, Pompetzki D, Raith AJ, Hauck SM, Amann B, Suppmann S (2006). Identification and functional validation of novel autoantigens in equine uveitis. Mol Cell Proteomics.

[3] Caspi RR, Roberge FG, Chan CC, Wiggert B, Chader GJ, Rozenszajn LA (1988). A new model of autoimmune disease. Experimental autoimmune uveoretinitis induced in mice with two different retinal antigens. J Immunol.

[4] Caspi RR, Roberge FG, McAllister CG CG, el-Saied M, Kuwabara T, Gery I (1986). T cell lines mediating experimental autoimmune uveoretinitis (EAU) in the rat. J Immunol.

[5] Degroote RL, Hauck SM, Kremmer E, Amann B, Ueffing M, Deeg CA (2012). Altered expression of talin 1 in peripheral immune cells points to a significant role of the innate immune system in spontaneous autoimmune uveitis. J Proteomics.

[6] Luger D, Caspi R (2008). New perspectives on effector mechanisms in uveitis. Semin Immunopathol.

[7] Chong WP, van Panhuys N, Chen J, Silver PB, Jittayasothorn Y, Mattapallil MJ (2015). NK-DC crosstalk controls the autopathogenic Th17 response through an innate IFN-gamma-IL-27 axis. J Exp Med.

[8] Luger D, Silver PB, Tang J, Cua D, Chen Z, Iwakura Y (2008). Either a Th17 or a Th1 effector response can drive autoimmunity: conditions of disease induction affect dominant effector category. J Exp Med.

[9] Deeg CA, Reese S, Gerhards H, Wildner G, Kaspers B (2004). The uveitogenic potential of retinal S-antigen in horses. Invest Ophthalmol Vis Sci.

[10] Eberhardt C, Amann B, Feuchtinger A, Hauck SM, Deeg CA (2011). Differential expression of inwardly rectifying K+ channels and aquaporins 4 and 5 in autoimmune uveitis indicates misbalance in Muller glial cell-dependent ion and water homeostasis. Glia.

[11] Hauck SM, Schoeffmann S, Amann B, Stangassinger M, Gerhards H, Ueffing M (2007). Retinal Mueller glial cells trigger the hallmark inflammatory process in autoimmune uveitis. J Prot Res.

[12] Hauck SM, Dietter J, Kramer RL, Hofmaier F, Zipplies JK, Amann B (2010). Deciphering membrane-associated molecular processes in target tissue of autoimmune uveitis by label-free quantitative mass spectrometry. Mol Cell Proteomics.

[13] Takahashi S, Muta K, Sonoda H, Kato A, Abdeen A, Ikeda M (2014). The role of cysteine 227 in subcellular localization, water permeability, and multimerization of aquaporin-11. FEBS Open Bio.

[14] Day RE, Kitchen P, Owen DS, Bland C, Marshall L, Conner AC (2014). Human aquaporins: regulators of transcellular water flow. Biochim Biophys Acta Gen Subj.

[15] Gorelick D, Praetorius J, Tsunenari T, Nielsen S, Agre P (2006). Aquaporin-11: a channel protein lacking apparent transport function expressed in brain. BMC Biochem.

[16] Yakata K, Tani K, Fujiyoshi Y (2011). Water permeability and characterization of aquaporin-11. J Struct Biol.

[17] Rojek A, Füchtbauer E-M, Füchtbauer A, Jelen S, Malmendal A, Fenton RA (2013). Liver-specific aquaporin 11 knockout mice show rapid vacuolization of the rough endoplasmic reticulum in periportal hepatocytes after amino acid feeding. Am J Physiol Gastrointest Liver Physiol.

[18] Atochina-Vasserman EN, Biktasova A, Abramova E, Cheng DS, Polosukhin VV, Tanjore H (2013). Aquaporin 11 insufficiency modulates kidney susceptibility to oxidative stress. Am J Physiol Renal Physiol.

[19] Salik D, Motulsky E, Gregoire F, Delforge V, Bolaky N, Caspers L, Perret J, Willermain F, Delporte C: Modification of aquaporin expression in response to fenretinide-induced transdifferentiation of ARPE-19 cells into neuronal-like cells. Acta Ophthalmol. 2016;94:e59-e67. (http://www.ncbi.nlm.nih.gov/pubmed/26389809). 10.1111/aos.12837 26389809

[20] Deeg CA, Ehrenhofer M, Thurau SR, Reese S, Wildner G, Kaspers B (2002). Immunopathology of recurrent uveitis in spontaneously diseased horses. Exp Eye Res.

[21] Bringmann A, Pannicke T, Grosche J, Francke M, Wiedemann P, Skatchkov SN (2006). Muller cells in the healthy and diseased retina. Prog Retin Eye Res.

[22] Deeg CA, Hauck SM, Amann B, Pompetzki D, Altmann F, Raith A (2008). Equine recurrent uveitis—a spontaneous horse model of uveitis. Ophthalmic Res.

[23] Eberhardt C, Amann B, Stangassinger M, Hauck SM, Deeg CA (2012). Isolation, characterization and establishment of an equine retinal glial cell line: a prerequisite to investigate the physiological function of Muller cells in the retina. J Anim Physiol Anim Nutr (Berl).

[24] Ehrenhofer MC, Deeg CA, Reese S, Liebich HG, Stangassinger M, Kaspers B (2002). Normal structure and age-related changes of the equine retina. Vet Ophthalmol.

[25] Deeg CA, Amann B, Hauck SM, Kaspers B (2006). Defining cytochemical markers for different cell types in the equine retina. Anat Histol Embryol.

[26] Tran TL, Bek T, Holm L, la Cour M, Nielsen S, Prause JU (2013). Aquaporins 6–12 in the human eye. Acta Ophthalmol.

[27] Xi H, Katschke KJ, Li Y, Truong T, Lee WP, Diehl L (2016). IL-33 amplifies an innate immune response in the degenerating retina. J Exp Med.

[28] Mizutani M, Gerhardinger C, Lorenzi M (1998). Müller cell changes in human diabetic retinopathy. Diabetes.

[29] Park JI, Yang SH, Lee JP, Yoo SH, Kim YS (2015). Genetic predisposition of donors affects the allograft outcome in kidney transplantation: single-nucleotide polymorphism of aquaporin-11. Kidney Res Clin Pract.

[30] Tchekneva EE, Khuchua Z, Davis LS, Kadkina V, Dunn SR, Bachman S (2008). Single amino acid substitution in aquaporin 11 causes renal failure. J Am Soc Nephrol.

[31] Schey KL, Wang Z, Wenke JL, Qi Y (1840). Aquaporins in the eye: expression, function, and roles in ocular disease. Biochim Biophys Acta.

[32] Madeira A, Fernandez-Veledo S, Camps M, Zorzano A, Moura TF, Ceperuelo-Mallafre V (2014). Human aquaporin-11 is a water and glycerol channel and localizes in the vicinity of lipid droplets in human adipocytes. Obesity (Silver Spring).

[33] Ishibashi K, Hara S, Kondo S (2009). Aquaporin water channels in mammals. Clin Exp Nephrol.

[34] Ishibashi K, Tanaka Y, Morishita Y (1840). The role of mammalian superaquaporins inside the cell. Biochim Biophys Acta.

[35] Inoue Y, Sohara E, Kobayashi K, Chiga M, Rai T, Ishibashi K (2014). Aberrant glycosylation and localization of polycystin-1 cause polycystic kidney in an AQP11 knockout model. J Am Soc Nephrol.

[36] Morishita Y, Matsuzaki T, Hara-chikuma M, Andoo A, Shimono M, Matsuki A (2005). Disruption of aquaporin-11 produces polycystic kidneys following vacuolization of the proximal tubule. Mol Cell Biol.

[37] Ikeda M, Andoo A, Shimono M, Takamatsu N, Taki A, Muta K (2011). The NPC motif of aquaporin-11, unlike the NPA motif of known aquaporins, is essential for full expression of molecular function. J Biol Chem.

[38] Agre P, Brown D, Nielsen S (1995). Aquaporin water channels: unanswered questions and unresolved controversies. Curr Opin Cell Biol.

[39] Okada S, Misaka T, Tanaka Y, Matsumoto I, Ishibashi K, Sasaki S (2008). Aquaporin-11 knockout mice and polycystic kidney disease animals share a common mechanism of cyst formation. FASEB J.

[40] Rojek A, Fuchtbauer EM, Fuchtbauer A, Jelen S, Malmendal A, Fenton RA (2013). Liver-specific aquaporin 11 knockout mice show rapid vacuolization of the rough endoplasmic reticulum in periportal hepatocytes after amino acid feeding. Am J Physiol Gastrointest Liver Physiol.

